# LEVERAGING NURSING ASSESSMENT DATA TO IDENTIFY FRAILTY INDICATORS OF HOSPITAL DISCHARGE DISPOSITION

**DOI:** 10.1093/geroni/igad104.2987

**Published:** 2023-12-21

**Authors:** Sarah Ser, Laurence Solberg, Ragnhildur Bjarnadottir, Robert Lucero, Mattia Prosperi, Urszula Snigurska, Brian Celso

**Affiliations:** University of Florida, Gainesville, Florida, United States; NF/SG VHS, Newberry, Florida, United States; University of Florida, Gainesville, Florida, United States; University of California, Los Angeles, Los Angeles, California, United States; University of Florida, Gainesville, Florida, United States; University of Florida College of Nursing, Gainesville, Florida, United States; University of Florida Jacksonville, Jacksonville, Florida, United States

## Abstract

Frailty among older adults is associated with higher morbidity and mortality rates and poorer hospital outcomes. Although frailty is not routinely assessed in the hospital setting, elements of frailty are captured in nursing assessments. The purpose of this study was to examine components of the Risk Analysis Index (RAI) captured in electronic health record (EHR) assessment data and their associations with discharge disposition. This was a retrospective observational study of EHR assessment data which included encounters of older adult patients (≥ 65 years) who were admitted from home to a medical/surgical unit of an academic hospital in North Central Florida between January 2012 and May 2021. The components of the RAI included in the study were sex, age, cancer, renal failure, heart failure, cognitive decline, unintentional weight loss, poor appetite, and shortness of breath at rest. Descriptive statistics were generated and compared between patients discharged to home versus those who were discharged to Post-Acute Care (PAC) facilities. Unadjusted associations were assessed using univariate logistic regression. Consistent with existing literature on frailty, all but one of the included RAI components (i.e., male sex) exhibited higher odds of discharge to a PAC facility with strong statistical support. Recognizing functional decline due to frailty is integral to the mission of improving patient safety. This study provides a preliminary proof of concept for leveraging existing assessment data in the EHR to capture frailty in older adults. Future studies of the overall predictive value of RAI with disposition are warranted.

